# Severe West Nile Virus Neuroinvasive Disease: Clinical Characteristics, Short- and Long-Term Outcomes

**DOI:** 10.3390/pathogens11010052

**Published:** 2022-01-02

**Authors:** Marija Santini, Sara Haberle, Snježana Židovec-Lepej, Vladimir Savić, Marija Kusulja, Neven Papić, Klaudija Višković, Ivana Župetić, Giovanni Savini, Ljubo Barbić, Irena Tabain, Marko Kutleša, Vladimir Krajinović, Tanja Potočnik-Hunjadi, Elizabeta Dvorski, Tamara Butigan, Gordana Kolaric-Sviben, Vladimir Stevanović, Lana Gorenec, Ivana Grgić, Filip Glavač, Armin Mehmedović, Eddy Listeš, Tatjana Vilibić-Čavlek

**Affiliations:** 1 Department for Adult Intensive Care and Neuroinfections, University Hospital for Infectious Diseases “Dr Fran Mihaljević”, 10000 Zagreb, Croatia; marko.kutlescha@gmail.com (M.K.); vkrajinovic@bfm.hr (V.K.); 2 School of Medicine, University of Zagreb, 10000 Zagreb, Croatia; flp.glavac@gmail.com (F.G.); tatjana.vilibic-cavlek@hzjz.hr (T.V.-Č.); 3 Institute of Emergency Medicine Krapina-Zagorje County, 49000 Krapina, Croatia; haberle.sara@gmail.com; 4 Department of Immunological and Molecular Diagnostics, University Hospital for Infectious Diseases “Dr Fran Mihaljević”, 10000 Zagreb, Croatia; szidovec@gmail.com (S.Ž.-L.); lgorenec@gmail.com (L.G.); ivanahobby@gmail.com (I.G.); 5 Poultry Center, Croatian Veterinary Institute, 10000 Zagreb, Croatia; v_savic@veinst.hr; 6 Department for Emergency Medicine, University Hospital for Infectious Diseases “Dr Fran Mihaljević”, 10000 Zagreb, Croatia; mkusulja@kusulja.com; 7 Department for Viral Hepatitis, University Hospital for Infectious Diseases “Dr Fran Mihaljević”, 10000 Zagreb, Croatia; papic.neven@gmail.com; 8 Department for Radiology and Ultrasound Diagnostics, University Hospital for Infectious Diseases “Dr Fran Mihaljević”, 10000 Zagreb, Croatia; viskovick@gmail.com (K.V.); ivazup33@gmail.com (I.Ž.); mehmedovic.armin302@gmail.com (A.M.); 9 OIE Reference Center for West Nile Disease, Department of Virology, Istituto Zooprofilattico Sperimentale “G. Caporale”, 64100 Teramo, Italy; gsavini@izs.it; 10 Department of Microbiology and Infectious Diseases with Clinic, Faculty of Veterinary Medicine of University of Zagreb, 10000 Zagreb, Croatia; ljubo.barbic@vef.hr (L.B.); vladostevanovic@gmail.com (V.S.); 11 Department of Virology, Croatian Institute of Public Health, 10000 Zagreb, Croatia; irena.tabain@hzjz.hr; 12 Department of Infectious Diseases, Varazdin General Hospital, 42000 Varazdin, Croatia; tanja.potocnik.h@gmail.com (T.P.-H.); ebidvorski@gmail.com (E.D.); tbutigan@gmail.com (T.B.); 13 Department of Infectious Diseases, General Hospital “Dr Tomislav Bardek”, 48000 Koprivnica, Croatia; gkolaric@net.hr; 14 Laboratory for Diagnostics, Croatian Veterinary Institute, Veterinary Institute Split, 21000 Split, Croatia; e.listes.vzs@veinst.hr

**Keywords:** West Nile virus, encephalitis, neuroinvasive disease, acute flaccid paralysis, critical care, outcome, modified Rankin scale, prognosis

## Abstract

West Nile Virus Neuroinvasive Disease (WNV NID) requires prolonged intensive care treatment, resulting in high mortality and early disability. Long-term results are lacking. We have conducted an observational retrospective study with a prospective follow-up of WNV NID patients treated at the Intensive Care Unit (ICU), University Hospital for Infectious Diseases, Zagreb, Croatia, 2013–2018. Short-term outcomes were vital status, length of stay (LOS), modified Rankin Scale (mRS), and disposition at discharge. Long-term outcomes were vital status and mRS at follow-up. Twenty-three patients were identified, 78.3% males, median age 72 (range 33–84) years. Two patients (8.7%) died in the ICU, with no lethal outcomes after ICU discharge. The median ICU LOS was 19 days (range 5–73), and the median hospital LOS was 34 days (range 7–97). At discharge, 15 (65.2%) patients had moderate to severe/mRS 3–5, 6 (26.0%) had slight disability/mRS 2–1, no patients were symptom-free/mRS 0. Ten (47.6%) survivors were discharged to rehabilitation facilities. The median time to follow-up was nine months (range 6–69). At follow-up, seven patients died (30.5%), five (21.7%) had moderate to severe/mRS 3–5, one (4.3%) had slight disability/mRS 2–1, six (26.1%) had no symptoms/mRS 0, and four (17.4%) were lost to follow-up. Briefly, ten (43.5%) survivors improved their functional status, one (4.3%) was unaltered, and one (4.3%) aggravated. In patients with severe WNV NID, intensive treatment in the acute phase followed by inpatient rehabilitation resulted in significant recovery of functional status after several months.

## 1. Introduction

West Nile Virus (WNV) is a re-emerging mosquito-borne virus, increasingly present in most European countries. Though most of the infected individuals are asymptomatic (80%) or develop a self-limited flu-like disease (20%), 1% will develop the severe form of West Nile Virus Neuroinvasive Disease (WNV NID), manifesting as meningitis, encephalitis, Acute Flaccid Paralysis (AFP), or a combination of those [1,2,3,4]. WNV meningitis is characterized by fever and signs of meningeal inflammation, such as nuchal rigidity, photophobia, and nausea and vomiting. WNV encephalitis is associated with prolonged altered mental status, seizures, or focal neurological signs. Patients with severe WNV encephalitis may present with stupor or coma [5]. Acute paralysis associated with WNV infection has been attributed to a poliomyelitis-like syndrome, myeloradiculitis, and Guillain–Barré Syndrome (GBS) [6]. WNV poliomyelitis with or without brainstem involvement is the most common neuromuscular manifestation of WNV infection, resulting in asymmetric paralysis. The motor neurons in the anterior horns and in the brainstem are the major sites of pathology responsible for neuromuscular signs; however, inflammation may also involve motor axons (polyradiculitis) and peripheral nerves (GBS) [7,8]. In comparison to patients with poliomyelitis-like syndrome, those resembling GBS have symmetric weakness with sensory loss [9].

Since patients with WNV NID may have prolonged Intensive Care Unit (ICU) stays with considerable long-term morbidity and mortality, increased utilization of healthcare services is often required in epidemic years [10,11]. The case fatality rate in WNV NID cases is approximately 10% [11].

The data on critical care issues related to WNV NID and long-term physical and functional outcomes are not well described. Several studies showed that age and underlying comorbidities are the main risk factors associated with adverse outcomes. In addition, patients admitted in the ICU often required prolonged invasive mechanical ventilation, with high complication rates and poor outcomes [12,13].

In Croatia, the first autochthonous clinical cases of WNV NID were reported in 2012 in eastern regions [14]. After that, sporadic cases and outbreaks were continuously recorded in continental counties until 2018 [15,16], whereas no human WNV infections were detected in 2019 and 2020 [17]. The largest outbreak occurred in 2018, with 54 confirmed WNV NID cases, and 7 cases of WNV fever [16]. The majority of Croatian WNV patients presented with meningitis and meningoencephalitis. However, some rare clinical presentations, such as WNV retinitis [18], cerebellitis [19], and cauda equina arachnoiditis [20], were also reported. In addition, a case report of WNV encephalitis associated with acute ST-elevation myocardial infarction was described during the 2018 outbreak [21].

Short-term follow-up studies have shown a high rate of persistent neurologic deficits in patients with WNV NID [5]; however, the long-term neurologic sequelae associated with WNV NID has not been well studied. This study aimed to analyze clinical characteristics, immunological response, as well as short- and long-term outcomes in Croatian patients with severe WNV NID.

## 2. Results

In a six-year period (2013–2018), 23 adult patients with WNV NID were treated at the Department of Intensive Care Medicine and Neuroinfectology, the 18-bed Intensive Care Unit (ICU), at the University Hospital for Infectious Diseases (UHID), Zagreb, which is a referral center for central nervous system infections in Croatia. These patients represent 42.4% of a total of 53 patients treated at the UHID for any form of WNV infection during the observed period. Patients’ demographic, epidemiological, and clinical characteristics are presented in Table 1.

Most of the patients were male (18/78.3%) with a median age of 72 (range 33–84) years. Comorbidities afflicted 22 (95.6%) patients, most frequently: arterial hypertension (19/82.6%), diabetes mellitus (9/39.1%), and coronary artery disease (4/17.8%). Three patients (13.0%) were immunocompromised due to kidney transplantation.

**Figure 1:**
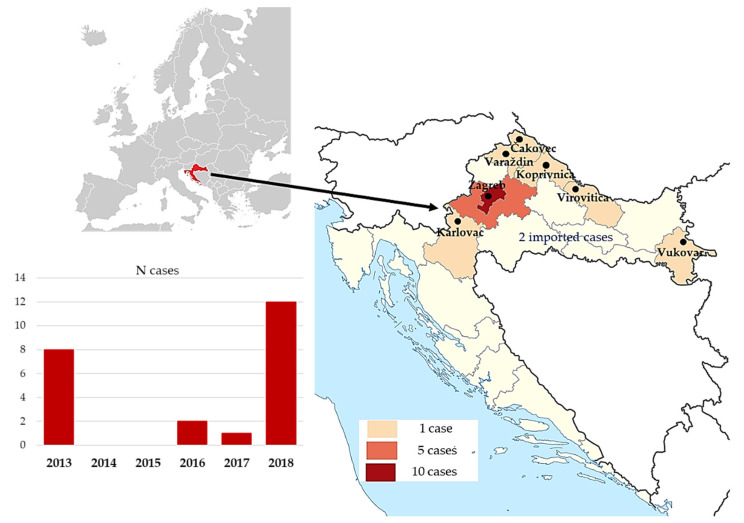
Distribution of patients with severe WNV NID in Croatia (2013–2018) according to year and geographic region.

The outbreaks of WNV infection striking Croatia resulted in 8 cases requiring ICU admissions in 2013, and 11 in 2018, whereas only individual cases were reported in the meantime (Figure 1). All patients were admitted between the second half of July and the first half of September.

Most of the patients originated from the City of Zagreb and Zagreb County (10/43.5% and 5/21.7%, respectively), whereas the remaining were referred from the other Croatian continental counties. There were two imported cases, in tourists coming from Hungary and the USA. None of the patients were previously vaccinated for Tick-Borne Encephalitis (TBE), yellow fever, or Japanese encephalitis.

Thirteen (56.5%) patients presented with encephalitis, and ten patients (43.4%) with encephalitis associated with Acute Flaccid Paralysis (AFP), whereas no patient had isolated meningitis or AFP. Fever, 39.3 ± 0.6 °C, was present in all patients. Headache was recorded in 10 (43.5%), whereas 18 (78.3%) patients had impaired consciousness at admission, with a median Glasgow Coma Scale (GCS) of 10 (range 3–15). The median duration of illness to hospitalization was four (range 1–13) days. Twelve (52.2%) patients were intubated and mechanically ventilated for a median duration of 12 days (range 5–73). In 2018, from 17 September to 9 October (for 22 days), six patients were on mechanical ventilation, representing the highest number of simultaneously mechanically ventilated patients, requiring 30% of ICU resources.

### 2.1. Laboratory Results

Routine CSF, hematological, and biochemistry results are presented in Table 2. All patients had elevated CSF cell count, median 197 (range 12–1529) cells/mm^3^. Seventeen patients (73.9%) had mononuclear cell predominance, median 60% (range 25–98). Mean CSF protein and glucose levels were 1.1 ± 0.5 g/L and 5.0 ± 2.1 mmol/L, respectively. All patients tested negative for syphilis and other frequent causes of encephalitis (herpes simplex virus 1 and 2, varicella-zoster virus, HIV, cytomegalovirus, Epstein–Barr virus, TBEV). CSF and blood cultures were all negative for bacterial pathogens. Biochemistry and hematology results were unremarkable.

### 2.2. Virology Results

**Figure 2:**
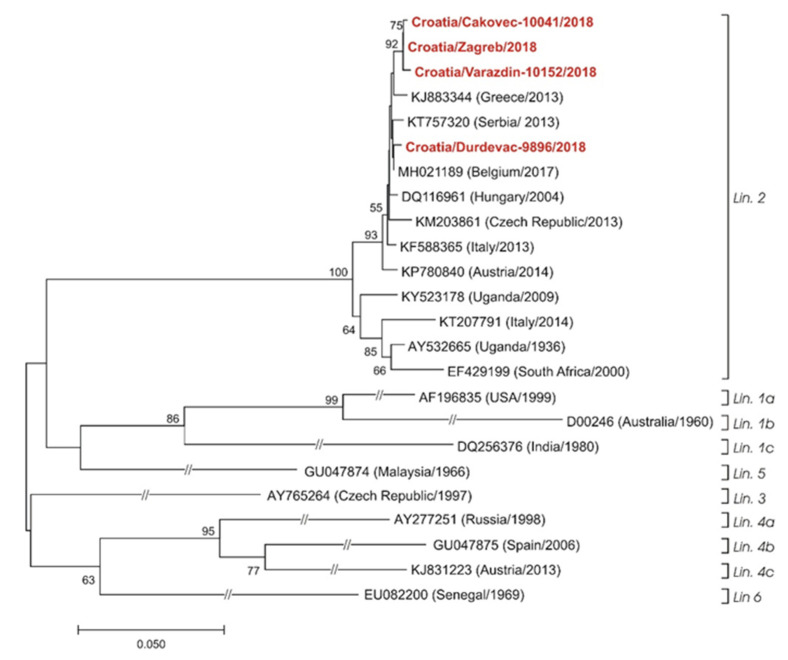
Phylogenetic neighbor-joining analysis of a 781-nucleotide fragment of the WNV NS5 gene (corresponding nucleotide positions 9076–9856 of the B956 strain, GenBank accession number AY532665) detected in patients with severe WNV NID in Croatia, 2018, and representative WNV strains. GenBank accession numbers, countries of origins, and isolation/detection years are indicated at the branches. Viruses from Croatia that were sequenced in this study are marked in bold and red color. WNV genetic lineages suggested by Rizzoli et al. (2015) [22] are indicated on the right. Lineage 7 could not be included in the analysis due to only partial sequence availability. Supporting (≥50%) bootstrap values of 1000 replicates are displayed at the nodes. Horizontal distances are proportional to genetic distance. Scale bar indicates nucleotide substitutions per site. The interrupted branches, indicated by double slashes, were shortened by 50% for better graphic representation.

In all patients, WNV IgM antibodies were demonstrated (median ratio 2.65, IQR = 1.29–3.82) in the period from 4 to 12 days after the onset of symptoms. WNV IgG antibodies were detected in 17 patients (median 47.61 RU/mL, IQR = 3.54–85.00). The IgG avidity was low in 15 patients (median avidity index 33%, IQR = 29–35), and borderline/high in two patients (40% and 86%, respectively). In six patients, WNV IgG seroconversion was documented in paired serum samples collected in the period from 7 to 14 days after the first sampling. WNV neutralizing antibodies were confirmed in eight patients (median titer 10, IQR = 5–20). WNV RNA was detectable in 17 tested samples: 4 serum, 3 CSF, and 10 urine samples. Nucleotide sequences were obtained for four strains detected in urine samples of patients with WNV NID. Phylogenetic analysis showed the circulation of the WNV lineage 2 (Figure 2).

### 2.3. Antiviral Cytokine Concentrations in Serum and CSF

Analysis of cytokine expression in the serum and CSF of patients with WNV infection collected from 6 to 15 days after disease onset revealed a characteristic pattern, presented in Table 3. The most prominent feature of intrathecal cytokine response to WNV was the presence of IL-6 in all samples, accompanied by the expression of IFN-γ in the majority of patients (9/11, 81.8%). Expression of IL-2, TNF-α, IL-17A, IL-17F, IL-4, IL-21, and IL-22 was limited to the serum, and with no positive CSF samples. Other cytokines, including IL-5, IL-13, IL-9, and IL-10, were detectable in the CSF of 1–3 patients only. Apart from the expression of IL-6 in the majority of serum samples (13/86.6%), other cytokines were present in the limited number of patients.

Although higher levels of IL-6 and IFN-γ were observed in the CSF (IL-6 median 6099.65 pg/mL, IQR = 5802.53–6190.50; IFN-γ median 127.37 pg/mL, IQR = 59.18–192.77) compared to serum (IL-6 median 220.52 pg/mL, IQR = 57.28–256.52; IFN-γ median 89.82 pg/mL, IQR = 38.19–138.41), the difference was significant only for IL-6 (Mann–Whitney U = 0, *p* < 0.001).

### 2.4. Neuroimaging and Electroencephalography Results

**Figure 3:**
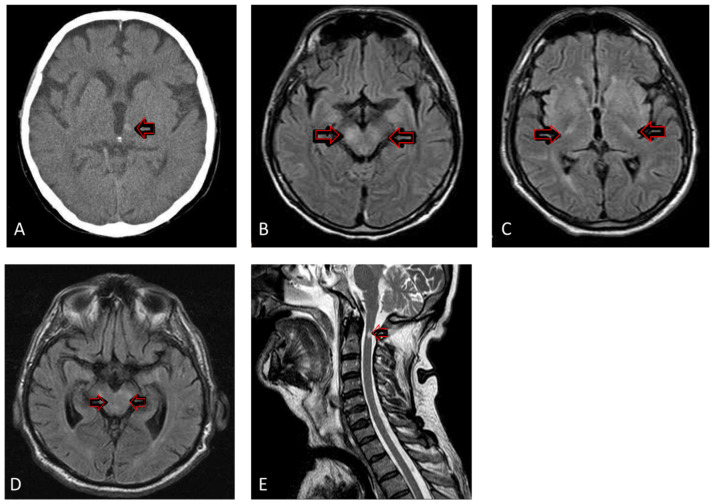
Selected brain and spinal cord Computer Tomography (CT) and Magnetic Resonance (MR) imaging in patients with severe WNV NID. (**A**): Native brain CT depicting a focal zone of haemorrhage in the left thalamus (arrow) (**B**): Brain MR in fluid attenuation inversion recovery (FLAIR) sequence in axial plane revealing hyperintense signal in crura cerebri (arrows) (**C**): Brain MR in FLAIR sequence in axial plane demonstrating high intensity signal in basal ganglia (arrows) (**D**): Brain MR in FLAIR sequence in axial plane displaying hyperintense signal in mesencephalon (arrows) (**E**): T2 weighted spinal MR, showing hyperintense signal in cervical spinal cord (arrow).

The most remarkable neuroimaging findings of the observed patient cohort are presented in Figure 3.

Magnetic Resonance (MR) imaging was performed in 10 (43.5%) patients. Seven patients had brain MR, and four had abnormal findings; two had inflammatory signs affecting crura cerebri bilaterally, accompanied by meningeal enhancement. One patient had inflammation signs in the pons and mesencephalon, and one in the cerebellum and basal ganglia. Three patients had brain and spinal cord MRs performed. The brain MR was unremarkable in all of them, whereas the spinal MR in two patients demonstrated abnormal findings: cervical myelitis in one, and cauda equina arachnoiditis in another, as we described in a previously published case report [20].

Fifteen (65.2%) patients were examined by brain CT, mainly before the lumbar puncture was performed, to exclude massive hemorrhages or other space-occupying lesions. Brain CT findings were normal in all patients, except one with a small focal hemorrhage in the left thalamus (Figure 3A).

Electroencephalography (EEG) was performed in 21 (91.3%) patients. The result was abnormal in 20 patients; 14 patients had diffuse slowing, whereas 6 had diffusely irregular EEG.

### 2.5. Outcomes of Patients with Severe WNV NID

**Figure 4:**
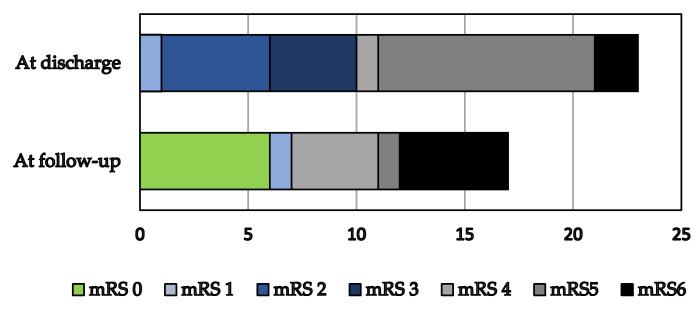
Outcomes at follow-up for 21 survivors. mRS Modified Rankin Scale: 0, no symptoms at all; 1, no significant disability despite symptoms, able to carry out all usual duties and activities; 2, slight disability, unable to carry out all previous activities, but able to look after own affairs without assistance; 3, moderate disability, requiring some help, but able to walk without assistance; 4, moderately severe disability, unable to walk without assistance, and unable to attend to own bodily needs without assistance; 5, severe disability, bedridden, incontinent, and requiring constant nursing care and attention; 6, death.

Short- and long-term outcomes are demonstrated in Table 4 and Figure 4.

Two patients (8.7%) died during the ICU treatment. Both patients were from the 2018 outbreak, and they presented with encephalitis. The first patient was an 84-year-old male with a history of arterial hypertension who died due to heart failure on the 25th hospital day. The second was a 73-year-old female with a history of arterial hypertension and atrial fibrillation who died due to ventilator-associated pneumonia on the 73rd day of hospitalization. There were no lethal outcomes during the hospital treatment after ICU discharge. The median ICU length of stay was 19 days (range 5–73), whereas the median hospital stay was 34 days (range 7–97). At discharge, there were 15 (65.2%) patients with moderate to severe disability according to the modified Rankin Scale (mRS 3–5), 6 (26.0%) patients with slight disability or minor symptoms persisting, and no patients without symptoms.

Information about seventeen (73.9%) patients was available for follow-up in July 2019. The median time to follow-up was nine months (range 6–69). Analyzing all the patients enrolled, five additional patients died during the follow-up, making the total number of patients with lethal outcomes seven (30.5%). However, data on the causes of lethal outcomes were not available. Five (21.7%) patients had moderate to severe disability (mRS 3–5), one (4.3%) slight or no disability (mRS 2–1), six (26.1%) patients had no symptoms (mRS 0), and four (17.4%) patients were lost to follow-up.

Summarizing mRS dynamics from the discharge to the follow-up, one (4.3%) patient aggravated, one (4.3%) was unaltered, and ten (43.5%) patients improved their functional status (six to no symptoms at all).

## 3. Discussion

This study indicates that severe WNV NID makes up a substantial proportion of all patients hospitalized for WNV infections in a Croatian referral center during the epidemic years. The observed patients presented equally with encephalitis and a combination of encephalitis with AFP, and required prolonged ICU and hospital treatment. Half of the patients were invasively mechanically ventilated. The ICU mortality rate, matching the hospital one, was relatively low (8.6%). Nevertheless, 60% of patients were deconditioned with moderate to severe functional status impairment, and needing further rehabilitation in specialized centers. During the follow-up period, 20% of survivors died, but half of the survivors experienced functional status improvement, whereas 28% had no symptoms. 

The results of this study are essentially accordant with the results of the studies examining the long-term recovery [23,24,25] and functional status of patients with severe WNV NID [12,26]. In our research and the research conducted in the USA [12], there were 65.2% and 76.9% patients with moderate to severe disability at discharge (mRS 3–5), respectively. In a 2012 cohort from Serbia, there were 9.6% of patients with moderate to severe disability, and 73.1% of patients with good functional status at discharge [9]. Nevertheless, in the study mentioned before, 15.4% of patients presented with meningitis, a milder form of WNV NID, whereas our study enrolled only patients with encephalitis, and a combination of encephalitis and AFP. Therefore, the difference in functional outcome at discharge among the mentioned studies can be explained by the varying severity of WNV NID enrolled. The common finding of our research and studies dealing with long-term outcomes is that intensive treatment in the acute phase of the WNV NID followed by medical rehabilitation can lead to excellent functional status after several months.

Mortality rate and other short-term outcomes are accordant with other studies focusing on short-term outcomes without functional status examination [13,27].

Our research and studies published so far have shown that severe WNV NID affects principally males and persons of advanced age, with common comorbidities, the most common being arterial hypertension and diabetes. We had fewer immunocompromised patients compared to Hawkes et al.: 13.0% and 44.0%, respectively. Regarding diverse gender predispositions for WNV NID and male predominance, it was speculated about different everyday male activities, possible longer stays outdoors, and inherent higher exposure to mosquito bites. However, so far, we cannot name an evidence-based and reasonable explanation of what makes males, and patients with diabetes and arterial hypertension prone to severe WNV NID, similar as in COVID-19 [28].

Epidemiological characteristics of patients in the observed cohort revealed WNV NID occurring only in the Croatian continental counties, spreading from east to west, with the highest number of cases in 2013 and 2018. The majority of patients originated from the Zagreb surroundings, Northwest Croatia. In addition, there were two patients with severe WNV NID coming from Hungary and the USA. The incidence of severe WNV NID follows WNV activity in Europe. Therefore, in some years, the incidence is low, or no cases occur. As in other studies, WNV demonstrated clear seasonal occurrence during the late summer and early autumn when Croatia’s tourism season peaks. The Croatian mainland is characterized by a continental climate with cold winters and hot summers. Average temperatures during summer are in the mid-to-high 20s °C. July and August are Croatia’s hottest months, with a temperature average of 24–25 °C. In addition, in summer and autumn 2018, when the largest Croatian outbreak occurred, absolute maximum air temperatures were above averages (0.7–3.3 °C in eastern, and 2.2–4.4 °C in north-western regions). In October 2018, when the last WNV cases were detected, thermal conditions were extremely warm (99 percentile) in the wider Zagreb area, and very warm (92–96 per centile) in other continental regions [16].

The clinical presentation of WNV NID is nonspecific. Patients with severe forms are admitted to the ICU relatively fast, after a median of four days (range 1 to 3 days) from the disease onset. Usual CNS inflammatory signs and symptoms were present: fever in all patients, headache in 40%, and impaired consciousness in almost 80%, with a median GCS of 10 (range 3–15). CSF analysis generally shows mononuclear pleocytosis, with moderately elevated protein levels and normal glucose levels. Other results of routine blood analysis, such as CRP, blood count, and liver enzymes, are nonspecific.

WNV lineage 2 has been regularly identified as the cause of local outbreaks and sporadic cases of infection in eastern and central Europe [29,30]. Four detected strains from the urine of Croatian patients with WNV NID showed circulation of WNV lineage 2, as well.

Despite a well-established role of proinflammatory cytokines and chemokines in the pathogenesis of WNV NID in in vitro and animal models, the data on their intrathecal expression, and their role in disease severity is limited to studies in pre-symptomatic and asymptomatic blood donors, and patients with post-infectious long-term sequelae [8,31,32,33,34,35]. Recently, we described the expression patterns of cytokines in paired CSF and serum samples collected from 37 WNV NID patients after a median of 5 days since the onset of symptoms, and in the serum of patients with WNV fever. A well-defined pattern of cytokine expression that included a high intrathecal synthesis of IL-6; lack of significant differences in the serum vs. CSF concentrations of IL-13, IL-9, IL-10, IFN-γ, and IL-22; as well as by the absence of IL-2, IL-4, TNF-α, and Th17 cytokines in the CSF was observed [36]. The present study results, focusing specifically on severe WNV NID, have shown a consistent expression of IL-6 in the CSF and serum. Further studies on the possible value of IL-6 as a neuroinflammatory biomarker or immunomodulation target in severe WNND seem to be warranted. 

Numerous in vitro studies and animal models have shown that IL-6 plays a vital role in the physiological homeostasis of neural tissues by enabling the differentiation of oligodendrocytes, the regeneration of peripheral nerves, and by acting as a neurotrophic factor [37]. In addition to its neuroprotective role, IL-6 has been implicated in the pathogenesis of autoimmune and inflammatory diseases, including multiple sclerosis, and Parkinson’s and Alzheimer’s disease. The biological background of these opposing observations is associated with two distinct molecular pathways used for IL-mediated signaling. The neuroprotective role of this cytokine is associated with the “classical pathway” mediated by the membrane IL-6 receptor on a restricted repertoire of cells (including microglia). In contrast, the proinflammatory role is associated with the “trans-signaling pathway”, mediated by an interaction between the IL-6/soluble IL-6R complex with the gp130 subunit, characterized by a ubiquitous expression on multiple cellular targets in the CNS [37]. The relative contribution of the neuroprotective and neuroinflammatory role of IL-6 in severe WNV NID is, at present, unknown. Therefore, considering the complexity of molecular pathways associated with IL-6-mediated signaling in vivo, the possible use of therapeutic strategies targeting the IL-6/IL-6R pathway (such as tocilizumab) or gp130-mediated pathways in WNND need to be carefully evaluated.

The neuroradiologic diagnostics were performed by MR in 43.5% of patients. Half of them had unremarkable findings, whereas the rest had inflammatory lesions at different localizations in the basal ganglia, cerebellum, and mesencephalon. Two of the three patients with spinal MR had an abnormal finding, comprising cervical myelitis and cauda equina arachnoiditis. Abnormal MR findings in patients with WNV NID have been reported in approximately one-third of cases [38]. Usually, these lesions are subtle, do not cause substantial mass effect, have no postcontrast enhancement, and do not correlate with the clinical severity of the disease. Pathologic signals on MR usually appear relatively late in the course of the disease (at average, from the second to the fourth week since the disease onset) [38]. Since the imaging findings are generally nonspecific, the differential diagnosis should consider Acute Disseminated Encephalomyelitis (ADEM), multiple sclerosis, white matter microvascular disease, and other viral encephalitides [38,39]. According to the research published to date, MR is performed in approximately half of the patients with WNV NID, and there is no predictive localization for detectable inflammatory lesions. This small proportion of patients with MR performed could be explained partially by the severity of the WNV NID itself, the need for mechanical ventilation, and the still limited conditions to perform MR for a mechanically ventilated patient. Most research dates to the early 2000s, and it would probably be worthwhile to examine future WNV NID patients with new MR technologies to better characterize this severe and debilitating disease. The literature describes eight patients with isolated cauda equina arachnoiditis, and three patients with spinal lesions accompanied by cauda equina arachnoiditis [9,40,41,42,43,44,45]. There is the unanswered question of whether WNV has a prediction for cauda equina. The only way to answer this question is detailed MR diagnostics in all patients with WNV NID.

Brain CT was mainly performed as a screening for space-occupying lesions before CSF sampling. As in other inflammatory CNS diseases, brain CT did not provide helpful information, except in detecting minor hemorrhagic cerebrovascular incidents [13].

Most patients with severe WNV NID have an abnormal, but nonspecific, EEG record: mainly, severe diffuse slowing, or diffuse irregularities.

According to the clinical presentation and laboratory findings, WNV NID is difficult to distinguish from other aseptic CNS inflammations: primarily, arboviral infections, such as TBE and Usutu virus encephalitis [15,46]. However, severe WNV NID ultimately presents with encephalitis in a combination of encephalitis with AFP, and AFP is more specific for WNV NID than other CNS infections.

This study has several limitations. First, nerve conduction studies and electromyogram tests were not performed because they were not available in hospital diagnostics. In addition, we were not provided with data on the functional status of patients before WNV NID. Finally, there is probably a referral bias, due to which there is a high proportion of severe WNV NID patients in the total number of those hospitalized for WNV infections.

However, this is a detailed presentation of severe WNV NID from its first occurrence in Croatia in 2013 to the last outbreak in 2018, with short-term and long-term outcomes analysis. The cytokine response documentation with a phylogenetic analysis of sequenced virus strains supports the main clinical results.

## 4. Materials and Methods

### 4.1. Patients

This observational retrospective study with prospective follow-up was conducted at the UHID, Zagreb, Croatia. We have searched the electronic database of the Department for Intensive Care Medicine and Neuroinfectology from January 2013 to December 2018. All patients treated at this department, aged ≥18 years, who met the criteria for WNV NID as defined in Table 5, were included in the study.

Patients with solid organ transplants, patients with malignant diseases receiving chemotherapy, and patients with autoimmune diseases treated by immunosuppressive therapy were considered immunocompromised. Following chart review, demographics, comorbidities, presentation of signs and symptoms, diagnostic tests, length of stay, mortality, and functional status measured by mRS were recorded. Follow-up was performed by telephone interview with patients or their caregivers in July 2019. Those patients who failed to respond to four calls during July 2019 were considered lost for follow-up.

Short-term outcomes were observed as vital status at discharge from the ICU and the hospital, ICU and hospital length of stay, mRS at discharge, and disposition.

For long-term outcomes, we evaluated vital status, mRS at follow-up, and changes of mRS from the discharge to the follow-up.

### 4.2. West Nile Virus Detection and Phylogenetic Analysis

Serum and CSF samples were collected from all patients, whereas urine samples were available for 14 patients. Samples were collected from 4 to 18 days after disease onset. CSF and urine samples were tested for the presence of neuroinvasive arboviruses using a Reverse Transcription-Polymerase Chain Reaction (RT-PCR): Tick-Borne Encephalitis, TBEV [47]; Usutu, USUV [48]; Toscana, TOSV [49]; Tahyna, TAHV [50]; and Bhanja virus, BHAV [51]. Additionally, serum and CSF samples were tested for the presence of IgM and IgG antibodies to TBEV/USUV (ELISA; Euroimmun, Lübeck, Germany) and TOSV (IFA; Euroimmun, Lübeck, Germany).

WNV RNA was extracted from serum, CSF, and urine samples using a High Pure Viral Nucleic Acid Kit (Roche Applied Science). A TaqMan real-time RT-PCR assay for the detection of WNV RNA was performed according to the protocol of Tang et al. (2006) [52]. Positive samples were subjected to conventional RT-PCR using PrimeScript™ One Step RT-PCR Kit Ver.2 (Takara Bio Inc, Kusatsu, Japan), and PanFlavi primers targeting the WNV NS5 gene (FP: 5′-TACAACATGATGGGVAARAGAGAGA-3′, RP: 5′-AGCATGTCTTCYGTBGTCATCCAYT-3′) to amplify a 1085 bp according to the protocol of Weissenböck et al. (2002) [53]. Amplified products were visualized on 1% agarose gel. DNA samples extracted from excised gel fragments were Sanger sequenced in both directions by Humanizing Genomics, Macrogen Inc. with the use of the same primers as for the RT-PCR. After sequencing, the raw nucleotide sequences were assembled, and the primer sequences were trimmed off. Genotyping and phylogenetic grouping of obtained sequences were based on a comparison with strains retrieved from the GenBank, and obtained using the BLAST algorithm (http://www.ncbi.nlm.nih.gov, accessed on 10 October 2021). Neighbor-joining phylogenetic analysis was conducted, and the evolutionary analyses were performed by using MEGA7 [54].

Serologic tests of CSF and serum samples (WNV IgM/IgG antibodies, IgG avidity) were performed using commercial enzyme-linked immunosorbent assays (ELISA; Euroimmun, Lübeck, Germany). Results were interpreted as follows: ELISA IgM ratio < 0.8 negative, 0.8–1.1 borderline, ≥1.1 positive; IgG relative units (RU/mL) <16 negative, 16–22 borderline, ≥16 positive. IgG positive samples were tested for IgG avidity using urea as a denaturing agent. The IgG avidity index (AI) was calculated and expressed as a percentage using the extinction values with and without urea treatment, and interpreted as follows: <40% low AI, 40–60% borderline AI, >60% high AI [55]. Initially reactive samples were confirmed using a VNT. The antibody titer was defined as the reciprocal value of the highest serum dilution that showed 100% neutralization. A titer of ≥10 was considered positive. Prior to VNT, WNV strain Eg-101 was titrated by 50% TCID (TCID50) using Vero cells. After four days, the titer was determined using the Reed and Muench formula [56].

### 4.3. Quantification of Cytokines in Serum and CSF

The analysis of cytokine expression in the CSF (*n* = 11) and serum (*n* = 15) of patients with WNV infection was performed by using multiplex bead-based flow cytometry on a FACS Canto II flow cytometer (Beckton Dickinson, USA). A cytokine panel LEGENDplex Human Th cytokine panel (BioLegend, San Diego, CA, USA) was used to analyze the expression of 13 cytokines, including those associated with innate and early proinflammatory immune responses (TNF-α, IL-6); Th1 type cytokine (IL-2, IFN-γ); Th2 cytokines (IL-4, IL-5, IL-9 and IL-13); Th17 cytokines (IL-17A, IL-17F, IL-21 and IL-22); and IL-10, the key anti-inflammatory cytokine [57]. Biological samples were stored at 80 °C until testing.

### 4.4. Statistical Analysis 

The frequencies are presented with 95% confidence intervals (CI). The cytokine levels in paired CSF and serum samples were compared using a Mann–Whitney U test. For statistical analysis, software package STATA/IC ver 11.2 (StataCorp LP, College Station, TX, USA) was used. The level of statistical significance was α = 0.05.

## 5. Conclusions

WNV NID is the most severe form of WNV infection, most commonly affecting elderly male patients, burdened with comorbidities, such as diabetes and arterial hypertension. The most common forms of this disease are encephalitis, and encephalitis associated with AFP. Although clinical and neuroradiological manifestations are nonspecific, WNV NID should be undoubtedly considered in the differential diagnosis of all patients with CNS infection during summer and autumn. During the epidemic years, WNV NID requires frequent ICU treatment. Although hospital mortality is not high, most patients have moderate to severe disability at discharge, and half of them need further inpatient rehabilitation. However, after several months, a substantial percentage of patients will recover to complete functional independence. We believe that future research will demonstrate the diagnostic and potentially therapeutic importance of cytokines, such as IL-6, which is significantly elevated in the CSF of these patients.

## Figures and Tables

**Table 1:** Demographic, epidemiological, and clinical characteristics of patients with severe WNV NID.

Characteristic.	*n* = 23 (% or Range)
Demographics	
Age, median (range) years	72 (33–84)
Male gender, *n* (%)	18 (78.3)
Area of residence	
Urban, *n* (%)	16 (69.6)
Suburban/rural, *n* (%)	5 (21.7)
Imported infections, *n* (%)	2 (8.7)
Patients with comorbidities	22 (95.6)
Arterial hypertension, *n* (%)	19 (82.6)
Diabetes mellitus, *n* (%)	9 (39.1)
Coronary artery disease, *n* (%)	4 (17.8)
Kidney transplant, *n* (%)	3 (13.0)
Date of admittance (range)	21 July–13 September
TBE/YF/JE vaccination	0 (0)
Clinical presentation	
Meningitis, *n* (%)	0 (0)
Encephalitis, *n* (%)	13 (56.5)
Encephalitis + AFP, *n* (%)	10 (43.5)
Isolated AFP, *n* (%)	0 (0)
Signs and symptoms at presentation	
Fever, *n* (%)	23 (100)
Fever (°C), mean ± SD	39.3 ± 0.6
Consciousness impairment, *n* (%)	18 (78.3%)
GCS, median (range)	10 (3–15)
Headache, *n* (%)	10 (43.5)
Tremor, *n* (%)	6 (26.1)
Diarrhea, *n* (%)	4 (17.4)
Days to hospitalization, median (range)	4 (1–13)
Mechanical ventilation, *n* (%)	12 (52.2)
Duration of mechanical ventilation, median days (range)	12 (5–73)

WNV NID = West Nile virus neuroinvasive disease; TBE = tick-borne encephalitis; YF = yellow fever; JE = Japanese encephalitis; AFP = acute flaccid paralysis; SD = standard deviation; GCS = Glasgow Coma Scale.

**Table 2:** Routine laboratory tests in patients with severe WNV NID.

CSF Results		
Patients with elevated CSF cell count, *n* (%)	23 (100%)	Reference values
Cells/mm^3^, median (range)	197 (12–1520)	0–5
Patients with CSF mononuclear predominance, *n* (%)	17 (73.9%)	
Mononuclear cells, % median (range)	60 (25–98)	Mononuclear 100%
Proteins (g/L) mean ± SD	1.1 ± 0.5	0.17–0.37 g/L
Glucose (mmol/L) mean ± SD	5.0 ± 2.1	2.5–3.3 mmol/L
Bacterial cultures and PCR	Negative (100%)	
Hematological and biochemistry results (average ± standard deviation)		
CRP (mg/L)	44.2 ± 45.9	<5.0 mg/L
White blood cell count (×10^9^/L)	11.17 ± 4.00	4.0–10.0 (×10^9^/L)
Red blood cells count (×10^12^/L)	4.45 ± 0.08	4.4–5.8 (×10^12^/L)
Hemoglobin (g/L)	133.26 ± 19.73	120–180 g/L
Platelets (×10^9^/L)	155.5 ± 58.35	100–400 (×10^9^/L)
AST (U/L)	37.13 ± 20.90	11–38 U/L
ALT (U/L)	34.9 ± 22.5	12–48 U/L
GGT (U/L)	73.9 ± 84.4	11–55 U/L

CSF = cerebrospinal fluid; CRP = C-reactive protein; AST = aspartate aminotransferase; ALT = alanine aminotransferase; GGT = gamma-glutamyl transferase.

**Table 3:** Cytokine response in serum and CSF samples of patients with WNV infection.

Cytokine	Serum (N = 15)		CSF (N = 11)	
	N Positive (%)	95% CI	N Positive (%)	95% CI
IL-5	4 (26.6)	7.8–55.1	1 (9.1)	0.2–41.3
IL-13	1 (6.6)	0.2–31.9	1 (9.1)	0.2–41.3
IL-2	1 (6.6)	0.2–31.9	0 (0)	0–28.5 *
IL-6	13 (86.6)	59.5–98.3	11 (100)	71.5–100 *
IL-9	3 (20.0)	4.3–48.1	3 (27.2)	6.0–60.9
IL-10	3 (20.0)	4.3–48.1	3 (27.2)	6.0–60.9
IFN-γ	10 (66.6)	38.4–88.2	9 (81.8)	48.2–97.7
TNF-α	4 (26.6)	7.8–55.1	0 (0)	0–28.5 *
IL-17A	4 (26.6)	7.8–55.1	0 (0)	0–28.5 *
IL-17F	2 (13.3)	1.6–40.5	0 (0)	0–28.5 *
IL-4	1 (6.6)	0.2–31.9	0 (0)	0–28.5 *
IL-21	2 (13.3)	1.6–40.5	0 (0)	0–28.5 *
IL-22	3 (20.0)	4.3–48.1	4 (36.3)	10.9–69.2

* One-sided 97.5% confidence interval.

**Table 4:** Outcomes of patients with severe WNV NID at discharge and at follow-up.

Outcomes at Discharge	
Lethal outcome in ICU, *n* (%)	2 (8.6)
Lethal outcome in hospital, *n* (%)	0 (0)
ICU length of stay, median days (range)	19 (5–73)
Hospital length of stay, median days (range)	34 (7–97)
Outcomes at follow-up	
Available at follow-up, *n* (%)	17 (73.9%)
Months to follow-up for survivors, median (range)	9 (6–69)
Deceased, *n* (%)	7 (30.4%)
Improved, *n* (%)	10 (43.5%)
Aggravated, *n* (%)	1 (4.3%)
Unaltered, *n* (%)	1 (4.3%)
Lost to follow-up, *n* (%)	4 (17.5%)

**Table 5:** Criteria for the WNV NID.

European Union clinical and laboratory criteria for WNV [40]. Laboratory diagnosis was confirmed by detecting WNV RNA in CSF (*n* = 3), WNV IgM in CSF (*n* = 5), WNV IgM/IgG in serum (*n* = 15), and confirmed by a Virus Neutralization Test (VNT), and 1 of the following:
Clinical evidence of meningitis (one or more of the following)
Positive meningeal signs
Pleocytosis in cerebrospinal fluid
Brain CT or MR finding consistent with inflammation
Clinical evidence of encephalitis (1 or more of the following)
Altered level of consciousness lasting at least 24 h
Focal neurological deficit
Seizure
Brain CT or MR finding consistent with brain inflammation
Abnormal electroencephalography
Acute-onset asymmetric limb weakness progressing over 48 h, and 2 or more of the following:
Hyporeflexia or areflexia of affected limbs
No pain or paresthesia of affected limbs
Pleocytosis ≥ 5 cells/mm^3^ , and elevated protein in cerebrospinal fluid
Spinal cord MR finding showing inflammation in anterior horns

## Data Availability

Not applicable.
